# Ultrasound evaluation in combination with finger extension force measurements of the forearm musculus extensor digitorum communis in healthy subjects

**DOI:** 10.1186/1471-2342-8-6

**Published:** 2008-03-03

**Authors:** Sofia Brorsson, Anna Nilsdotter, Marita Hilliges, Christer Sollerman, Ylva Aurell

**Affiliations:** 1 PRODEA research group, Halmstad University, Halmstad, Sweden; 2 R & D centre Spenshult Hospital for Rheumatic Diseases, Halmstad, Sweden; 3 Department of Hand Surgery, Sahlgrenska Academy, Göteborg, Sweden; 4 Department of Diagnostic Radiology, Halmstad Central Hospital, Halmstad, Sweden

## Abstract

**Background:**

The aim of this study was to evaluate the usefulness of an ultrasound-based method of examining extensor muscle architecture, especially the parameters important for force development. This paper presents the combination of two non-invasive methods for studying the extensor muscle architecture using ultrasound simultaneously with finger extension force measurements.

**Methods:**

M. extensor digitorum communis (EDC) was examined in 40 healthy subjects, 20 women and 20 men, aged 35–73 years. Ultrasound measurements were made in a relaxed position of the hand as well as in full contraction. Muscle cross-sectional area (CSA), pennation angle and contraction patterns were measured with ultrasound, and muscle volume and fascicle length were also estimated. Finger extension force was measured using a newly developed finger force measurement device.

**Results:**

The following muscle parameters were determined: CSA, circumference, thickness, pennation angles and changes in shape of the muscle CSA. The mean EDC volume in men was 28.3 cm^3 ^and in women 16.6 cm^3^. The mean CSA was 2.54 cm^2 ^for men and 1.84 cm^2 ^for women. The mean pennation angle for men was 6.5° and for women 5.5°. The mean muscle thickness for men was 1.2 cm and for women 0.76 cm. The mean fascicle length for men was 7.3 cm and for women 5.0 cm. Significant differences were found between men and women regarding EDC volume (p < 0.001), CSA (p < 0.001), pennation angle (p < 0.05), muscle thickness (p < 0.001), fascicle length (p < 0.001) and finger force (p < 0.001). Changes in the shape of muscle architecture during contraction were more pronounced in men than women (p < 0.01). The mean finger extension force for men was 96.7 N and for women 39.6 N. Muscle parameters related to the extension force differed between men and women. For men the muscle volume and muscle CSA were related to extension force, while for women muscle thickness was related to the extension force.

**Conclusion:**

Ultrasound is a useful tool for studying muscle architectures in EDC. Muscle parameters of importance for force development were identified. Knowledge concerning the correlation between muscle dynamics and force is of importance for the development of new hand training programmes and rehabilitation after surgery.

## Background

The architecture of skeletal muscles is a primary determinant of muscle function [1,2]. Knowledge of muscle architecture is of great practical importance in understanding the relation between muscle structure, force and exertion ability [3]. Muscle architecture is mainly characterized by fascicle length, pennation angle and the thickness of the muscle [4-6]. Knowledge of human muscle architecture has until recently been based on the dissection of cadaver specimens or biopsies. However, the morphological characteristics of muscles in embalmed cadavers have been reported to change due to shrinkage. Furthermore, neither cadaver muscles nor biopsy specimens allow muscle fibre morphology and force development to be studied during contraction in the living human being [7]. Martin et al. pointed out that the architectural characteristics of muscle differ between cadaver muscle and *in vivo *muscle, both in relaxed and contracted conditions [8]. Hence, non-invasive methods on living subjects are required to study muscle contraction patterns and real-time muscle changes in architecture during force development. By using ultrasound (US) it is possible to obtain detailed, dynamic information on the muscle architecture non-invasively. In previous US studies of the lower and upper extremities, parameters such as fascicle length, muscle cross-sectional area, shape changes, muscle thickness, muscle volume and pennation angles have been shown to affect the manner in which muscle force is transmitted to the tendons and bones [9-11].

US has been used in several studies to provide information about the morphological structure of different muscles. In their US studies Shi et al. combined US with surface electromyography for detecting muscle architectural changes in muscles during fatigue and acting for feasibility of prosthesis [12,13]. Aagaard et al. used US to measure the response to strength training and the changes in muscle architecture [2]. US has also been used to study the differences between men and women regarding muscle parameters such as muscle pennation angles and muscle fascicle length [14]. US has been applied to the rotator cuff muscles to analyse the dynamic contraction pattern of these muscles to confirm the neuromuscular intensity [15]. Fukunaga et al. used US to measure muscle architecture and function in human muscles. They pointed out that the use of cadavers for studies of architecture and modelling of muscle functions would result in inaccurate and, in some cases, misleading results [1].

Grip function is based on the force of the muscles involved in finger and wrist motion. A sometimes neglected but, important ability in ensuring good hand function is wrist and finger extension motion. Finger extension control is one of the most difficult motions to regain after disease/injury and is also very important for prehensile activities. Indeed, loss of this capability is a primary disabler for hand function [16]. The force that can be generated is dependent on the muscle architecture, including aspects such as muscle fibre length, muscle pennation angle, the contraction pattern, muscle thickness and muscle volume [17-19].

In order to assess how disease influences muscle morphology and function, it is necessary to establish baseline knowledge concerning normal forearm muscles. To the best of our knowledge, no ultrasound-based architectural studies have been performed in combination with extension force measurements on the extensor muscles controlling hand function. This study was therefore conducted to investigate the relation between architectural parameters of the forearm and extension force *in vivo *in healthy subjects, based on measurements of the m. extensor digitorum communis (EDC).

The two specific aims of the study were:

a) to identify parameters describing the architecture of the EDC using ultrasound, and

b) to investigate the relationship between these muscle parameters and finger extension force in healthy men and women.

## Methods

### Subjects

The EDC was examined in 40 test subjects, 20 men and 20 women (Table 1). The test subjects were matched for age and sex and had similar occupations (office work). Their dominant hands were selected for the investigation. Individuals with inflammatory diseases and hand or arm injuries were excluded. The purpose of the study and the experimental procedures were explained to all the subjects before they gave their written consent to participate. The investigation was approved by the by the ethics committee at Lunds University and all procedures complied with the Declaration of Helsinki.

**Table 1:** Subject characteristics as mean values ± SD (range)

	Men	Women
Age (years)	54 ± 9.1 (35–68)	58 ± 10.6 (36–73)
Height (cm)	185.0 ± 7.5 (168–190)	166.5 ± 5.4 (158–174)
Weight (kg)	88 ± 6.5 (70–120)	68 ± 5.4 (54–80)

### Measurement equipment

All US examinations were performed with a Siemens Acuson Aspen system using a 7.5 MHz linear transducer (38 mm width). The dynamic image was recorded digitally as cine-loops.

**Figure 1:**
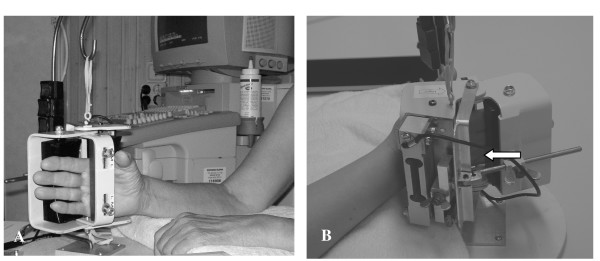
**Finger extension force measurement device**. (A) The device used to measure finger extension force. (B) The arrow shows the placement of theMCP joints.

A recently developed finger extension force measurement device was used to measure the finger extension force. This new device was designed specifically for the fingers based on the biomechanics of the hand (Fig. 1) and provides data for the whole hand, as well as single-finger extension forces, expressed as maximal force, mean force and continuous force over a specified time [20,21]. Measurements can be made at different angles, α, of the metacarpophalangeal (MCP) joint, although all the results presented in this paper were obtained at the same angle [22]. The design of the device ensures identical positioning of the hand on each examination. Evaluation and validation of this new finger extension force measurement device will be published shortly elsewhere.

### Finger extension force measurements in combination with US

The procedure for the finger extension measurements was standardized in terms of sitting position, instructions and encouragement [23,24]. All measurements (force measurements and US) were conducted by the same investigator (SO). The sitting position was that recommended by the American Society of Hand Therapists [25]. The subjects were seated in an upright position on a chair in front of the instrument, with their feet flat on the floor. Their forearm rested on a supporting pillow and their hand was placed in the extension measurement device, which was positioned on a table in front of them, with the other hand resting on the table. The wrist was not immobilized, and the joint angle was in a neutral position (0–30 degrees extension) during the measurements. The shoulder was adducted and neutrally rotated, while the elbow joint had approximately 90° flexion [26]. The subjects were instructed to extend and press their fingers as hard as possible against the resistance of the hand pad in the extension device.

**Figure 2:**
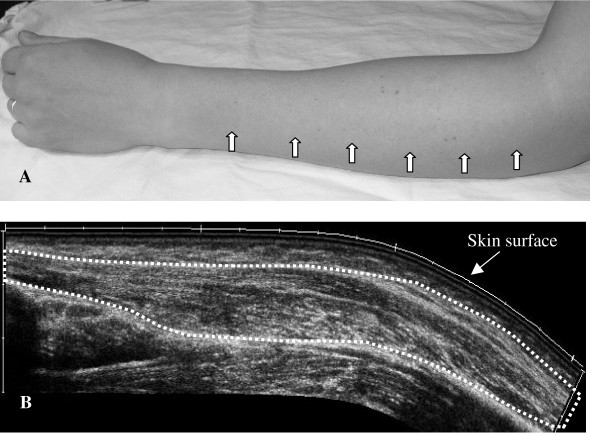
**Extended file of view**. (A) The measuring points (arrows) on themuscle used to calculate the CSA and the volume of the EDC. (B) US image obtained using the extended field of view technique, illustrating the EDC in the longitudinal plane (dotted line).

Before the US measurements, the subject's forearm was measured, and six points were marked on the arm (Fig. 2). The subjects were seated as described above, US images of the hand were recorded in the relaxed position and while performing the finger extension force measurements. Ultrasound transmission gel (AQUASONIC^® ^100) was used for US imaging. Ultrasound images were obtained from each subject on one occasion.

### Muscle parameters measured with US

**Figure 3:**
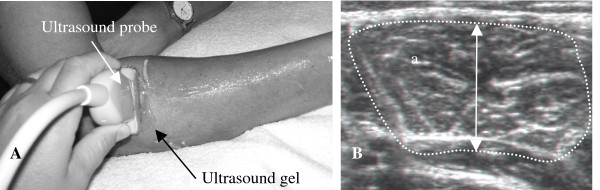
**US measuring point**. (A) Position of the probe for ultrasound measurements of the EDC, 15% distal of the EDC origin. (B) Transverse US image obtained at the measurement position (contracted muscle). The circumference is shown by the dotted line, the cross-sectional area is the area within the line, and the muscle thickness is indicated by the double-headed arrow.

Limb lengths were measured using anatomical landmarks: underarm length, and the distances between the olecranon process of the ulna and the processus styloideus of the ulna. For measurement purposes, the live US images (cine-loops in the transverse and longitudinal planes) were reviewed and measurements were carried out on the still US image of the completely relaxed muscle, as well as the fully contracted muscle (live cine-loops). The optimal and standardized location for US measurements was a point distal from the origin of the EDC (the lateral epicondyle) corresponding to 15% of the total arm length (Fig. 3). This location exhibited the largest muscle area, which was clearly defined and thus easy to measure, and is referred to as the measuring point in the text.

The following parameters were measured: muscle circumference, muscle thickness, muscle cross-sectional area (CSA), muscle volume, pennation angle, contraction pattern and fascicle length. These parameters are believed to be important for force development. In addition, EDC distal muscle-tendon contraction was measured.

#### a) Anthropometry measurements of the forearm, muscle circumference, muscle thickness, muscle CSA and muscle volume

Anthropometry measurements of the length of the ulna, defined as the distance between the olecranon process of the ulna and the processus styloideus of the ulna, were made carefully. The two landmarks were obtained with US and the ulna was then measured with a measuring tape. The muscle CSA, muscle circumference and muscle thickness were measured on a still US images in the transverse plane at the measuring point. EDC volumes were calculated by summing the six cross-sectional areas, each of which was multiplied by the respective interslice distance. Measurements were made on saved images at different levels of the EDC [2]. These cross-sectional areas were interspaced by a distance of 3 cm, starting at 15% distal from EDC origin. Five measurements were made, at 30, 45, 60, 75 and 90% of the ulna length (Fig. 2) [27].

#### b) Muscle fibre pennation angle

**Figure 4:**
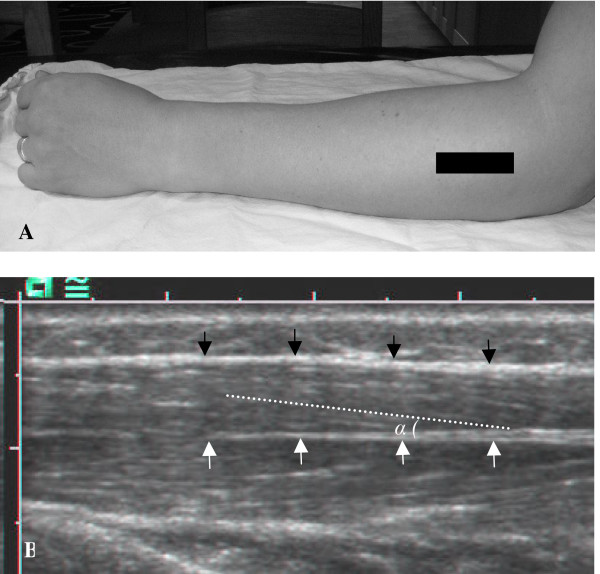
**Muscle pennation angle**. (A) The black rectangle shows the position of the US probe during pennation angle measurements. (B) The longitudinal US image showing the superficial aponeurosis (black arrows), the deep aponeurosis (white arrows) and the pennation angle (α).

The pennation angle was defined as the angle created by the fascicles and the insertion into the deep aponeurosis. Longitudinal US images were recorded at 15% distal from the muscle origin, according to procedures described previously [2]. The pennation angle was measured as the angle between the muscle fibres and the deep aponeurosis of the insertion of the tendon when the fingers were extended (Fig. 4).

#### c) Contraction pattern

**Figure 5:**
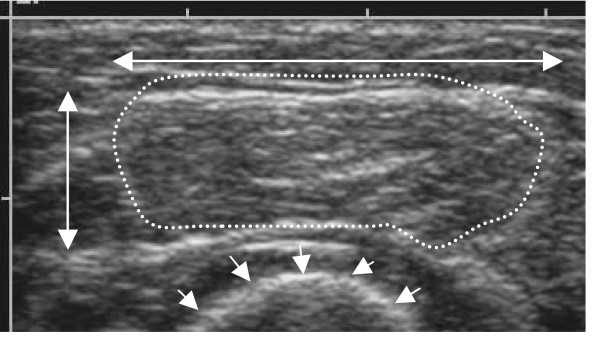
**Muscle shape changes**. Transverse US image used to obtain muscleheight (h) and muscle base (b) to calculate the change in shape of the muscle CSA. The olecranon ulna is indicated by the white arrows.

The contraction pattern was defined by two descriptors: the change in the shape of the muscle CSA (the relation between the length and diameter of the muscle) (Fig. 5), and the movement of the deep aponeurosis. Dynamic images were recorded at the measuring point in a transverse view to determine the change in muscle shape and the change in the position of the deep aponeurosis.

#### d) Fascicle length

The fascicle length was estimated from the muscle thickness and the pennation angle (α). The transducer was held parallel to the deep aponeurosis in the longitudinal plane in the position that best depicted the deep aponeurosis and was thus slightly oblique to the muscle fibres. The distance between the subcutaneous adipose tissue-muscle interface and the inter-muscular interface in the cross-sectional image was defined as the muscle thickness. The pennation angles were measured as described above (Fig. 4). The fascicle length was then estimated from the equation [28]:

*Fascicle length *= *muscle thickness *× *α*^-1^

#### e) Range of motion in the distal tendon of the EDC

**Figure 6:**
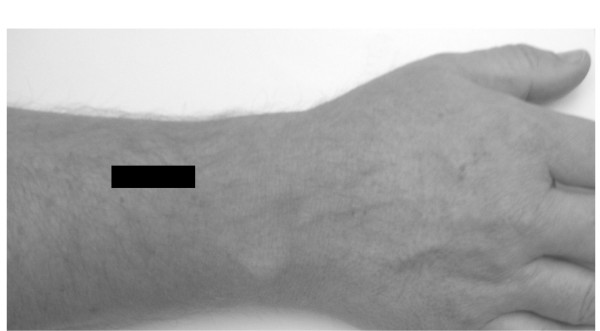
**Range of motion in distal tendon**. The black rectangle illustrates the position of the US probe when measuring the ability of the muscle toproduce a range of motion.

Three different approaches were taken to measure the distal insertion tendon position in the relaxed vs. the contracted muscle. Longitudinal and trans-sectional measurements at the level of the processus styloideus were evaluated (Fig. 6).

### Intra- and inter-observer agreement

All US images were interpreted blindly by two independent investigators (Observer I (SO) and Observer II (YA)) to establish the inter-observer agreement in the measurements. In order to estimate intra-observer agreement all the images were interpreted twice by one of the investigators (Observer I).

### Statistics

For group comparisons of independent samples the Mann-Whitney U-test was used. Descriptive data included means ± 1 SD. To assess the correlations between the measured variables, Spearman's rank (r_s_) correlation test was applied. A p-value of less than 0.05 (two-tailed test) was considered to be significant. Repeatability and agreement of the continuous variables were assessed using the graphic technique described by Bland and Altman [29]. Cohen's kappa was used for discrete variables in evaluating intra- and inter-observer agreement. Interpretation of the kappa value was based on the guidelines proposed by Landis & Koch [30]. SPSS version 11.5 for Windows XP and MedCalc 5.0 were used in the statistical analysis.

## Results

### Muscle architecture parameters in the EDC

In US imaging it is necessary to have well defined anatomic landmarks in order to establish reliable reference points for measurements. No major difficulties were encountered in assessing the muscle circumference (data not shown), muscle CSA, muscle thickness or pennation angles. The anatomical landmark used to define the measurement point for this parameter was quite easy to find. However, no reliable landmark was obtained for measurements of the range of motion in the EDC distal tendon during muscle contraction, and because of this, consistent results could not be obtained with the equipment and method used. No difficulties were encountered in assessing the changes in shape of the CSA. Rotation of the muscle was observed at the deep aponeurosis during contraction, but could not been measured with the methods used.

### Muscle architecture parameters in relation to force

**Figure 7:**
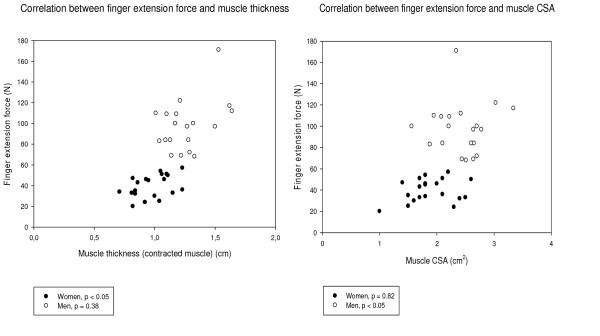
**Correlation finger extension force – CSA and finger extension force – muscle thickness**. Muscle thickness (left), and muscle CSA (right), as a function of finger extension force for each individual.

The extension force showed a strong correlation to contracted muscle thickness for women (r_s _= 0.47, p < 0.05) but not for men (p = 0.38) (Fig. 7). Two correlations were found with extension force for men: muscle volume (r_s _= 0.58, p < 0.01) and muscle CSA (r_s _= 0.48, p < 0.05). No correlation was found between extension force and muscle shape change (men, p = 0.79, women, p = 0.30) or extension force and fascicle length (men, p = 0.60, women, p = 0.65). No correlations were found between extension force and muscle volume (p = 0.70) or between extension force and muscle CSA for women (p = 0.82). Extension force and pennation angle showed no correlation in either of the groups (men, p = 0.38, women, p = 0.49). When the data from men and women were analysed together, finger extension force was strongly correlated to muscle volume (r_s _= 0.85, p < 0.01), muscle thickness (contracted muscle) (r_s _= 0.71, p < 0.01), muscle CSA (r_s _= 0.53, p < 0.01) and change in muscle shape (r_s _= 0.38, p < 0.05).

### Differences in muscle architecture parameters between healthy men and women

There was a significant difference between the muscle anatomy of men and women. The mean length of the forearm in men was 23.1 ± 1.0 cm (range 22–25) and in women 21.1 ± 1.2 cm (range 20–22). The mean EDC volume in men was 28.3 ± 4.8 cm^3 ^(range 18.6–43.1) and in women 16.6 ± 4.6 cm^3 ^(range 9.7–28.9) (see Fig. 4). The mean CSA for men was 2.54 ± 0.4 cm^2 ^(range 1.6–3.3) and for women 1.9 ± 0.4 cm^2 ^(range 1.0–2.6). The mean fascicle length for men was 7.3 ± 2.4 cm (range 3.8–10.8) and for women 5.0 ± 0.9 cm (range 3.9–7.0). The mean muscle thickness for men was 1.1 ± 0.2 cm (range 0.7–1.5) and for women 0.8 ± 0.1 cm (range 0.6–1.1). The pennation angle for men was 6.6 ± 1.3 cm (range 3.5–8.5) and for women 5.7 ± 1.4 cm (range 4.08.5). Significant differences were found between men and women regarding muscle volume (p < 0.001), CSA (p < 0.001), fascicle length (p < 0.001), muscle thickness (p < 0.001) and pennation angle (p < 0.05). The overall shape changes in muscle CSA during contraction were more pronounced for men than for women, (p < 0.01). The finger extension force for men was 97.9 ± 24.0 N (range 68–171) and for women 39.6 ± 10.8 N (range 20 – 57) (p < 0.001).

### Evaluation of US measurements

To be able to evaluate the inter- and intra-observer agreement in the interpretation of the dynamic images were assessed regarding CSA, pennation angle and muscle thickness. The intra-observer agreements are expressed as the mean kappa value for the two repeated measurements, and the inter-observer agreements as the mean kappa value for the two investigators (Table 2). The mean inter-observer difference in CSA was 0.22 ± 0.2 cm^2 ^for men and 0.08 ± 0.2 cm^2 ^for women. The difference in the mean value of the pennation angle was -0.2 ± 0.7° for men and -0.3 ± 0.9° for women. The mean inter-observer difference in muscle thickness was 0.03 ± 0.07 for men and 0.05 ± 0.08 for women.

**Table 2:** Intra- and inter-observer agreement expressed as kappa values

	CSA (cm^2^)	Pennation angle (°)	Thickness (cm)
Intra-observer agreement (men)	0.90	0.80	0.89
Inter-observer agreement (men)	0.81	0.84	0.86

Intra-observer agreement (women)	0.92	0.81	0.85
Inter-observer agreement (women)	0.83	0.84	0.75

**Figure 8:**
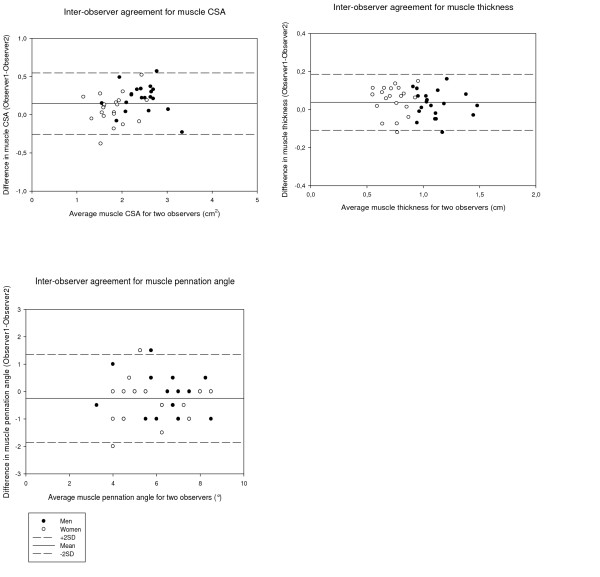
**Inter-observer agreement between Observer I and Observer II**. Inter-observer agreement in measurements of muscle CSA, muscle thickness and muscle pennation angles for each subject.

The distributions of the differences and limits of agreement for 95% of the cases obtained from the inter-observer assessment of the three parameters are presented in Fig. 8. The mean intra-observer difference for CSA was 0.10 ± 0.1 cm^2 ^for men and -0.04 ± 0.1 cm^2 ^for women. The difference in the mean value of the pennation angle was -0.4 ± 0.5° for men and -0.33 ± 0.8° for women. The mean intra-observer difference for muscle thickness for men was -0.05 ± 0.1 cm and for women 0.03 ± 0.04 cm.

## Discussion

Ultrasound in combination with finger extension force measurement is non-invasive techniques that can be used to dynamically study functionally important muscle parameters. Muscle architecture can be identified and quantified with US allowing for a more thorough understanding of the muscle architecture and its relation to force. This knowledge is of importance in understanding functional correlations involved in generating force, which is a prerequisite for scientifically based hand training/rehabilitation programmes.

US has been used previously in several studies to measure muscle architecture. In this study we focused on the EDC in healthy men and women. To our knowledge, no other *in vivo *imaging study has been presented describing the EDC muscle architecture in men and women, and its relation to finger extension force.

### Muscle architecture parameters in the EDC

This study revealed significant differences between the muscle architecture in men and women. The men had a larger muscle volume, muscle CSA, muscle thickness, longer fascicles and larger pennation angles. The EDC is a bipennate muscle where the range of motion and power generated by the muscle depend on the arrangement of the fascicles. Furthermore, bipennate muscles have been observed to be very powerful [31]. The range of motion in the distal tendon and the rotation in the EDC could not be measured. However, it would be of great interest to measure these, since it is likely that these parameters are important in developing force. Kawakami et al. showed in their study (on the m. medial gastrocnemius, lateral gastrocnemius and m. soleus) how the muscle parameters changed during contraction, and suggested that the changes could reflect the muscles' ability to produce force [32]. In further research on the EDC it would be of interest to analyse how this muscle responds during contraction at different locations of the muscle. This could provide information about the muscle as well as the elastic characteristics of the aponeurosis and tendon. It is also possible that the EDC, a muscle designed for precision tasks and grip control rather than force exertion, is constructed differently from the large force-generating muscles in the lower limbs.

### Muscle architecture and force generation

Architectural differences between muscles are claimed to be the best predictors of force generation [7]. Several muscle architectural parameters are theoretically related to force, and decreases in muscle volume, muscle CSA, muscle pennation angle and muscle fascicle length are regarded as being important causes of declining strength [33,34]. However, in the present study no correlation was found between finger extension force and fascicle length. One reason for this could be the geometric method used to calculate the fascicle length. The method is based on several assumptions, for example, that the fascicles are straight. It is generally accepted that a close relation exists between the CSA of a muscle and its ability to generate force [35,36]. The present study revealed a correlation between muscle CSA and finger extension force for men but not for women. These results are partly in agreement with previous studies. Fukunaga et al. found a relationship between isometric arm flexion force and the CSA of the arm flexor muscle, and their conclusion was that arm force is proportional to the CSA [17]. The present findings suggest that muscle strength is related to muscle volume for men but not for women. However, a correlation was found between muscle volume and finger extension force in the present study when the data from the two groups were pooled together (rs = 0.85, p < 0.01). Trapper, Holzbaur and colleagues used magnet resonance imaging to study the relationship between muscle volume and force. They found correlations between muscle volume and muscle force in both the upper and lower extremities. However, their study groups were small (n = 18 and n = 10) and the data from men and women were not analysed separately [37-39].

In an experimentally impressive study, Zuurbier and Huijing measured the muscle pennation angles (in the medial gastrocnemius in rat) using small wire markers on the muscle surface, which were filmed during contraction. The results of their study showed that the pennation angle varied considerably in the muscle [40]. Two other research groups have made significant contributions in the area of fibre rotation during muscle shortening. Rotation allows muscle fibres to maintain a higher level of force than if they are constrained to maintain a constant pennation angle [4,32]. The results of the present study support previous findings concerning muscle rotation during contraction. Fibre rotation was observed as well as muscle shape changes. However, no correlation was found between the change in shape of the muscle and the extension force when analysing the data from men and women separately. Chi-Fishman et al. studied the change in m. rectus femoris and found a correlation between force capacity and muscle shape change. They compared patients with myositis and healthy controls [41].

In contrast to previous studies on differences between the sexes, the finger extension force for women in the present study was only 40% of the men's, although differences of 50–60% could be expected. Åstrand et al. reported that women had 60% of men's force in the upper extremities [42]. Grip strength is often recorded by clinicians as a quick and reliable/practical measure of hand impairment and function, and provides a useful measure of hand status. However, the hand is a complex structure, and for a total evaluation of hand function, grip strength in combination with non-invasive evaluation method such as US can provide more knowledge about the extensor muscles and the effects on this muscles and the hand function after rehabilitation.

In the present study, we found differences in the correlations between muscle architecture and extension force in men and women. These could be due to the experimental set-up or methodological problems, although a large effort was made to standardize the procedures and we have demonstrated good validity and reproducibility of the methods in this, and in previous studies. Another explanation could be that the study population was too small to detect correlations. However, this is not very likely since there is not even a statistical tendency towards significance. Yet another possibility could be that there is a breakpoint, in other words, the muscle has to have a specific size before any correlation is seen between muscle volume and force, and muscle CSA and force. This would explain why the men in this study showed a correlation between force and muscle volume and CSA, but not the women. It must also be noted that muscle properties are not alone in determining muscle function, also the neural-muscle interaction influence the movement pattern and force production of the muscles [43].

### Methodological considerations

Various methods can be used to study muscle architecture, including ultrasound, magnetic resonance imaging [2,44] and laser diffraction [45]. Laser diffraction is an invasive technique, while magnetic resonance imaging is only suitable for static measurements. Ultrasound, on the other hand, is non-invasive and clearly shows the movement of the muscle [1]. It is also harmless, can easily be repeated and offers the possibility of dynamic examinations.

Bland and Altman's graphical method was used to study the agreement between Observer I and II [29]. The graphs show the differences between each pair of measurements plotted against their mean, with one line showing the mean of the differences, and two more lines representing two SD above and below the mean. The graphs must be interpreted in related to the clinical situation, and the acceptable difference in measurements. Intra- and inter-observer kappa values for the muscle parameters were good to excellent. Our results regarding kappa analysis are in the same range as in previous studies [15,46,47]. In order to make a complete evaluation of the repeatability of the ultrasound method, the whole ultrasound investigation ought to be repeated by another examiner. However, this was not possible within the frame of this project.

## Conclusion

Ultrasound is a useful tool for studying functionally important muscle parameters.

The combination of two non-invasive methods provides new and detailed information concerning the EDC architecture and its relation to extension force. Baseline values concerning the EDC in healthy men and women, and the differences between the sexes regarding muscle parameters relevant for force generation have been presented. This knowledge may be of importance in the development of new methods for hand training and rehabilitation.

## Competing interests

The author(s) declare that they have no competing interests.

## Authors' contributions

SO participated in the study design, performed the finger force measurements, ultrasound measurements, statistical analysis, and prepared the draft of the manuscript. AN, MH, YA and CS took part in the study design, and contributed to the data evaluation and manuscript preparation. All authors read and approved the final manuscript.

## Pre-publication history

The pre-publication history for this paper can be accessed here:


